# Toward a culture of care in music higher education: setting the scene for collective well-being with a salutogenic, whole-institution approach

**DOI:** 10.3389/fpsyg.2025.1729483

**Published:** 2025-11-27

**Authors:** Veronika J. Lubert, Dawn C. Rose, Elena Alessandri

**Affiliations:** School of Music, Lucerne University of Applied Sciences and Arts, Lucerne, Switzerland

**Keywords:** salutogenesis, sense of coherence, resources, tertiary music education, health promotion measurement, music students

## Abstract

The current academic discourse in musicians’ health and well-being is dominated by pathogenic and preventive paradigms focusing primarily on risk reduction but underlining the urgent need for intervention, especially in music higher education. Efforts to implement various treatment and preventive strategies as well as health education and promotion curricula have so far yielded inconclusive results. We therefore propose a paradigm shift from pathogenesis to salutogenesis, emphasizing the factors that promote health and well-being. With this paper, we aim to provide an overview of the current landscape of musicians’ health research, discuss conceptual foundations, and outline our position of advocating for a theory-driven, salutogenic, whole-institution approach that should foster a culture of care and build on the design of a new, unified measurement tool to facilitate implementation and evaluation. Based on an emerging line of research on the role of challenges in salutogenic, resource-oriented approaches to music students’ health and well-being, we argue that a culture of care could include multiple stakeholders in music higher education and would contribute to health and well-being as an integral part of performance and success.

## 1 Introduction

Previous research on musicians’ health and well-being has provided consistent evidence documenting the physical and mental occupational hazards associated with professional music-making (Elam et al., 2022; Kegelaers and Gibney, in press; Rodríguez-Gude et al., 2023; Rotter et al., 2020). Consequently, the current academic discourse in this area is dominated by pathogenic and preventive paradigms focusing primarily on risk reduction but underlining the urgent need for intervention, especially in music higher education (MHE). Higher education music institutions (HEMIs; i.e., music universities, academies, faculties, colleges, and conservatories) have made efforts to implement various treatment and preventive strategies as well as health education and promotion curricula–with mixed results (Evans et al., 2024). Recent global guidelines for health and well-being in MHE have pressed for moving beyond individualistic approaches and instead suggested addressing the systemic and cultural conditions that shape musicians’ experiences (Matei et al., 2025).

To advance such cultural change while also supporting the individual in their personal responsibility toward health and well-being, we propose a paradigm shift from pathogenesis to salutogenesis, emphasizing the factors that promote health and well-being. We aim to provide an overview of current musicians’ health research, discuss conceptual foundations, and advocate for a theory-driven, salutogenic, whole-institution approach that should foster a culture of care and build on the design of a new, unified measurement tool to facilitate implementation and evaluation.

## 2 The current landscape of musicians’ health research

Most studies have used a pathogenic lens, that is, they have focused on the mechanisms of disease development by investigating the prevalence of physical problems and mental ill-health and associated risk factors. According to several large-scale studies, musicians report higher levels of mental ill-health, greater disturbance from work environments, and lower perceived general health than the general population (Vastamäki et al., 2023; Vermeersch et al., 2023). Research on the physical problems of musicians has tended to focus on playing-related musculoskeletal disorders (PRMDs; Rodríguez-Gude et al., 2023; Rotter et al., 2020). Large-scale studies have consistently shown many musicians experience PRMDs, with symptoms often intensifying during the transition into MHE (Cruder et al., 2020; Gembris et al., 2018, 2020). Additionally, studies have identified a range of physical health concerns, including hearing disorders (Hake et al., 2024), vocal strain (Childs et al., 2022; Pestana et al., 2017), substance abuse (Berg et al., 2022; King et al., 2024), and focal dystonia, a task-specific, neurological disorder with the potential to end musicians’ careers (Détári and Altenmüller, 2025).

The status of musicians’ health and well-being was reported as especially acute following the Covid-19 pandemic, during which many musicians endured such financial and psychological hardship that they eventually opted to change their careers (Shaughnessy et al., 2022; Spiro et al., 2021). These challenges, however, did not emerge solely during the pandemic; depression, general anxiety, and music performance anxiety have long been documented as the primary mental health concerns among musicians (Kegelaers and Gibney, in press). A recent study of a clinical sample of professional musicians additionally reported psychiatric diagnoses of adjustment, somatoform, and personality disorders (Fernholz et al., 2025). Mental health issues tend to be associated with poor physical health—though in which direction is not yet known—and linked to financial strain and career uncertainty, especially for students in MHE (Bruyneel et al., 2024; Perkins et al., 2017).

Consequently, HEMIs have made efforts to support students, and research has explored various curricula and strategies to reduce risks and enhance protective factors. Yet, evidence on the efficacy of these programs remains inconclusive (Evans et al., 2024; Laseur et al., 2023; Stanhope et al., 2022). Despite reported engagement in preventive strategies, these were often not integrated into daily routines, and many students appeared disengaged from their health—either knowingly or unknowingly (Spahn et al., 2017). These results suggest the need for a stronger focus on implementation.

While most of the literature has been focused on reducing risk factors, some studies have also investigated protective factors. Dispositional optimism (Orejudo et al., 2017) and having harmonious rather than obsessive passion for one’s art (Bonneville-Roussy and Vallerand, 2020) can act as a buffer against music-related anxiety. Harmonious passion as well as experiencing flow states have been associated with higher well-being and quality of life (Bonneville-Roussy and Vallerand, 2020; Habe et al., 2019). As the experience of flow is also negatively related to music performance anxiety, facilitating flow may be especially protective (Antonini Philippe et al., 2022; Cohen and Bodner, 2019a,b).

## 3 Conceptual foundations

Derived from the definitions of (mental) health by the World Health Organization (1948, 2022) and the concept of health for performance as opposed to general health (J. Ginsborg personal communication, May 20, 2025), we define musicians’ health as a state of complete physical, mental, and social well-being that enables musicians to constructively meet the challenges of their artistic everyday life, to practice and perform effectively, to develop their skills and talents, and to make a meaningful contribution to their community^1^. Adopting the definition of well-being as “the balance point between one’s resource pool and the challenges faced” (Dodge et al., 2012, p. 230), it becomes clear that we need to consider music students’ individual resources just as much as the challenges they face. The pathogenic lens has brought to light the many challenges of being a professional musician and highlighted the urgency of paying more attention to musicians’ health and well-being, especially in the context of MHE. To complement this perspective, a salutogenic lens would allow us to understand what positively contributes to musicians’ well-being, and what promotes and maintains their health.

Salutogenesis is a model explaining the relationship between health, stress, and coping by identifying the resources that help people to be—and stay—healthy (Antonovsky, 1979). At its core, one’s sense of coherence (SOC) is defined as the degree to which one perceives the world as comprehensible, manageable, and meaningful. In general, a stronger SOC enables individuals to preserve and develop their health when under stress. This can be achieved by using generalized resistance resources (GRRs)—the qualities of a person, such as optimism or harmonious passion, a collective (e.g., supportive peer group or ensemble), or a situation (e.g., a stable environment)—that facilitate “successful coping with the inherent stressors of human existence” (Antonovsky, 1996, p. 15). For example, musicians facing a high-stakes performance might draw on their self-efficacy and self-regulation capacities, support from fellow performers, and structured practice environments to manage stress effectively. Health promotion would thus be driven by enhancing one’s SOC and ensuring the availability of useful GRRs.

According to Antonovsky (1996), the strength of one’s SOC is influenced by one’s life experiences, that is, by their consistency, by how one navigates a balance between overload and underload, by the extent to which one feels consistent emotional bonds and belonging in social groups and to which one can participate in socially valued decision-making. Importantly, adopting a salutogenic perspective means considering the whole lifespan—a perspective that can have a particular impact for developing curricula in MHE where students have commonly already mastered their instrument (or voice) and where MHE can be considered the final training for a professional career. Salutogenesis as a philosophical underpinning of health promotion offers an overarching umbrella for several theories and concepts, including, but not limited to, self-efficacy, self-esteem, self-actualization, self-determination, and positive psychology (Eriksson and Contu, 2022). A salutogenic umbrella can also be meaningfully related to other theoretical approaches emphasizing musicians’ resources, adaptation, and development (Batt-Rawden, 2024; López-Íñiguez and Bennett, 2020).

## 4 Previous studies employing a salutogenic perspective on musicians’ health

Few studies in music have—explicitly or implicitly—been based on a salutogenic framework. Here, we discuss those that focused on protective factors mentioned under the salutogenic umbrella (Eriksson and Contu, 2022) and associated with musicians’ positive health outcomes. Research with professional musicians has foregrounded the role of music itself as a resource for their psychological health, providing affective experiences, feelings of belonging and support for mood regulation (Saarikallio et al., 2020). A study investigating the PERMA model from positive psychology with its five dimensions of well-being—positive emotions, engagement, relationships, meaning, and accomplishment—in professional musicians found higher scores for positive emotions, relationships, and meaning compared to the general population (Ascenso et al., 2018). According to Ascenso et al. (2017), the social structures in group music-making provide intrinsic value, such as shared memories of success and a group identity. To our knowledge, the PERMA model has not yet been directly applied to music students. With salutogenesis as an overarching framework, it could be better understood how PERMA alongside other positive psychological traits such as optimism, gratitude, or curiosity function and exert their effect on musicians’ well-being (Joseph and Sagy, 2022).

A study of music students’ vitality has further underpinned the promotive function of adaptability and the quality of peer relationships (Miksza et al., 2021). Looking at positive health factors for music teachers, researchers found that applying a more pedagogical approach, providing agency over their work situation, and drawing energy from the interplay of music and teaching were crucial for well-being (Fjellman-Wiklund et al., 2002). In a resource-oriented intervention study with professional musicians and music students, guided imagery seemed to be particularly beneficial for strengthening participants’ professional identity and nurturing their personal and professional resources (Trondalen, 2016).

Explicitly referring to salutogenesis, recent qualitative work explored health challenges for professional and student musicians. The study demonstrated the importance of actively managing one’s health and maintaining strong social support networks but also practicing with a sense of joy and enrichment, cultivating meaningfulness through experiencing musical highlights, and enhancing one’s coping capabilities when self-musicking (Batt-Rawden, 2024; Ege et al., 2023; Wang and Welch, 2024). Music students attributed most of their health challenges to performance pressure and requested integrated health education to help them establish healthy habits (Ege et al., 2023). Their personal and social resources included situational understanding, regular physical activity, motivation through meaning, and the sharing of music and synergy created between themselves and the audience. This emerging line of research illustrates the role of challenges in salutogenic, resource-oriented approaches to music students’ health and well-being: learning how to navigate challenges and stressors also contributes to developing one’s resistance resources and strengthening one’s SOC across one’s life course (Kirkland, 2025). At the same time, a resource-oriented approach should include boundary setting, advocacy, and, where appropriate, collective action to address unsafe or inequitable conditions, rather than simply adapting to them.

## 5 Position: a salutogenic, whole-institution approach toward a culture of care

**FIGURE 1:**
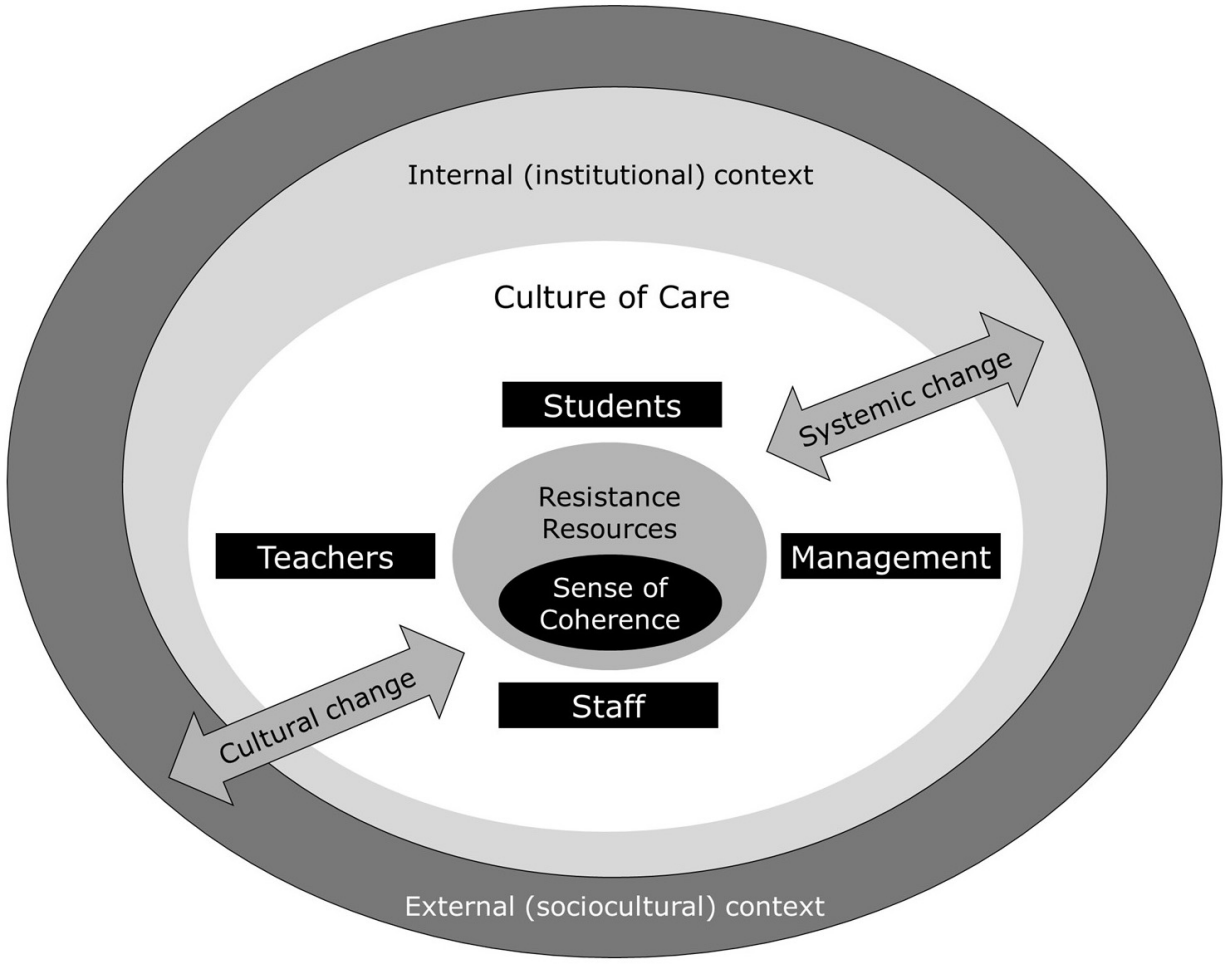
Key stakeholders and contextual factors involved in taking a holistic, salutogenic approach to supporting musicians’ health and well-being.

In advocating for a salutogenic, whole-institution approach, we distinguish between systemic change, referring to institutional structures, policies, and resource allocation, and cultural change, encompassing shared values, beliefs, and everyday practices within the institution’s community. Because the salutogenic model relates GRRs not only to qualities of an individual but also to situations and collectives (Antonovsky, 1996; Idan et al., 2022), it provides a suitable framework within which to foster systemic and cultural changes. While these two dimensions are deeply interdependent, systemic interventions can help initiate reform, whereas lasting impact depends on a corresponding cultural transformation that embeds health and well-being into the lived experience of students and staff. The innovative work by Shoebridge and Osborne (2025) shows how crucial it is to involve students in the conceptualization of such initiatives—in addition to other MHE stakeholders—and that truly effective health promotion must extend beyond education and support services to address the institutional culture itself (Matei et al., 2025). As institutions shift away from the master-apprentice model (Almqvist and Werner, 2024), consulting with students as key stakeholders offers valuable insights into how to develop and embed health and well-being into their curriculum. To better understand musicians’ health and well-being from a salutogenic perspective with the long-term goal to induce systemic and cultural changes in MHE, we suggest exploring the sense of coherence and GRRs for each of the four stakeholder groups: students, teachers, staff, and senior management. To support all actors in the system, their needs, challenges, values and beliefs should be considered alongside a contextual element, which includes both internal (institutional) and external (socio-cultural, healthcare, and inter-institutional) resources (see Figure 1).

While the salutogenic orientation has previously been used in whole-system approaches to health promotion in a variety of non-musical settings such as organizations, schools, higher education, and workplaces in general, the literature on single settings often “only loosely refers to salutogenesis” (Bauer, 2022, p. 277). Settings have been defined as the social and physical contexts of everyday life where environmental, organizational, and personal factors interact to shape health and well-being (Nutbeam and Kickbusch, 1998). Overall, research on the relationships between distinct settings and specifications of GRRs and SOC has been limited, except for intervention research to promote salutogenesis (Bauer, 2022). In higher education (HE), some studies indicate that a stronger sense of coherence is positively associated with health-promoting behaviors, self-related health and social support, as well as lower acculturative stress among international students (Dooris et al., 2022). A recent scoping review of whole-institution interventions in HE did not seem to have found any studies explicitly combining whole-institution approaches with salutogenesis (Sweeting et al., 2023). Consequently, future research on such a combination may yield significant potential for health promotion not just in MHE but HE more generally.

## 6 Discussion

That a holistic approach is necessary—emphasizing both personal responsibility and the influence of HEMIs through collective values, beliefs, and actions, including an ethical obligation to support sustainable careers—is not a new position (Chesky et al., 2006). Healthy Conservatoires Network (HCN) (2025) acknowledges this holistic view through an eight-factor wellness framework, encompassing emotional, environmental, financial, intellectual, occupational, physical, social, and spiritual dimensions. HCN also promotes a settings-based, whole-system approach to embed health and well-being in artistic and academic programs—instilling healthy habits, empowering all stakeholders, engaging participatory decision-making, and applying evidence-based research. In Australia, Wijsman and Ackermann (2019) proposed a translational approach, advocating for universal health education for music students, ongoing evaluation of initiatives, and the development of e-platforms to support institutional resources and global access. They emphasized institutions’ duty of care and the need for a cultural shift toward settings-based health literacy to prevent injury and promote lifelong well-being (Baadjou et al., 2019). While the above principles are promising, they may lack a clearly articulated theoretical foundation, making systematic evaluation difficult. The salutogenic model may provide the necessary foundations to develop a culture of care, thereby affording sustainable health promotion in MHE.

### 6.1 Principles of a culture of care

Recognizing that individuals are differently situated and that institutions bear collective responsibility for enabling care, the ethics of care do not rely on consensus but acknowledge care as an ongoing, contested process of interpreting and responding to needs (López-Íñiguez and Westerlund, 2023). In MHE, such an ethic implies a systemic capacity to reflect on and reorganize institutional practices—not merely to support the individual well-being of stakeholders, but to transform the institution’s understanding of its social purpose and its positioning within broader cultural frameworks. “Caring for, about, and with music students” thus involves more than momentary responsiveness; it requires a deep understanding of students’ diverse trajectories, motivations, and ways of engaging with feedback (Hendricks and McPherson, 2023, p. 417). On the one hand, teachers may benefit from developing “student literacy” (Hendricks, 2018), which refers to a reflective and empathetic capacity to “read” students and to co-construct learning environments grounded in trust and relational awareness. On the other hand, students should similarly be encouraged to communicate their needs and perspectives, requiring self-reflection, confidence and communication skills, which could be supported by educating them on “how to be a student” in the particular one-on-one setting in MHE (M. Robinson personal communication, July 2, 2025). Senior management could mirror these ideas in their leadership style, policy design, and resource allocation, whereas staff could ensure flexible and empathetic practices and building bridges helping to coordinate care across academic, artistic, and support services.

### 6.2 Implementation pathways guided by a salutogenic measurement tool

To translate a salutogenic, whole-institution approach into institutional reality, HEMIs must embed care and SOC into their structures, relationships, and pedagogical practices and foster GRRs. Strengthening SOC involves increasing comprehensibility through transparent communication and coherent policies, ensuring manageability by providing adequate resources and support, and cultivating meaningfulness through shared purpose and inclusive engagement. Evaluation may take various forms if we want to understand how actions toward a culture of care relate to musicians’ health and well-being. The pathogenic lens and its measurement tools of ill-health, for example for playing-related pain, musculoskeletal symptoms, anxiety and depression, appear limited as they do not capture the systemic, relational, and developmental dimensions that shape students’ and staff’s experiences. To complement rather than replace this lens, we propose the development of a salutogenic measurement tool—one that can help institutions assess and strengthen their capacity to promote health and well-being across all stakeholder groups.

To ensure contextual relevance and validity, we aim to develop the tool through an interdisciplinary and participatory process involving educators, researchers, health professionals, students, staff, and institutional leaders. Insights from an ongoing institutional ethnography in MHE (Sabo et al., 2024) could further inform this process. Once developed, the tool could guide senior management in identifying strengths and gaps in well-being support, assist educators in designing SOC-enhancing learning environments, and empower students and staff to co-create well-being initiatives.

## Data Availability

The original contributions presented in this study are included in this article/supplementary material, further inquiries can be directed to the corresponding author.

## Footnotes

1 This definition refers to any music-related capacity, including being a conductor, music teacher, DJ, composer, music therapist, etc.
